# Analysis of stiffness and damping performance of the composite leaf spring

**DOI:** 10.1038/s41598-022-11055-5

**Published:** 2022-04-27

**Authors:** Xiaojun Zou, Bao Zhang, Guodong Yin

**Affiliations:** 1 School of Cyber Science and Engineering, Southeast University, Nanjing, 211189 China; 2 Naveco Automobile Co., Ltd, Nanjing, 211806 China

**Keywords:** Composites, Mechanical properties

## Abstract

Lightweight design of leaf springs is conducive to reducing fuel consumption and improving vehicle comfort. The weight of leaf spring can be reduced obviously by using composite material. Stiffness and damping are the key factors that affect the properties of the leaf spring. The influence of the glass fiber laying angle and volume content on the stiffness and damping of the composite leaf spring was analyzed through experiment and simulation. The results show that the stiffness and damping properties of the leaf springs are related to the fiber laying angle and the fiber volume content. When the volume content and the number of layers are constant, the stiffness shows a nonlinear decreasing relationship with the laying angle, and the damping coefficient increases linearly with the laying angle. When the laying angle and the number of layers are constant, the stiffness increases linearly with the fiber volume content; the damping coefficient has a nonlinear decreasing relationship with the fiber volume content. The type of research can provide theoretical basis and reference for the design, analysis and optimization of composite leaf spring.

## Introduction

Due to the needs of environmental protection, energy saving and emission reduction, lightweight has become the current trend of automobile development. In addition, lightweight vehicles can also improve power, comfort, save materials and reduce costs^1^. Automobile lightweight technology is the integrated application of design, material and manufacturing technology. The two main ways to achieve lightweight are structural optimization design and the application of new materials. Compared with the steel of the same structure, using composite materials can greatly reduce the weight, especially the development of low-density composite materials provides more potential for automobile lightweight^2^. Among many composite materials, glass fiber resin matrix composites have the characteristics of lower density, higher strength and stiffness, good elasticity and corrosion resistance, etc. And the composite materials have been widely used in aerospace, automotive industry, machinery manufacturing and other fields^3,4^. Most of the elastic elements in the suspension system of commercial vehicles are leaf springs. However, the weight of the leaf spring is large and the damping performance is poor, which is not conducive to the lightweight and comfort of the vehicle. In order to reduce the weight of the suspension and improve the damping performance of suspension, the composite leaf spring have become the main research object^5,6^.

As one of the effective methods to reduce vibration and noise of automobile chassis, glass fiber reinforced composite leaf spring has received extensive attention in recent years. Ke et al.^7^ introduced the method of leaf spring design method, stiffness calculation and optimization. Guduru et al.^8^ developed a kind of glass fiber epoxy resin monolithic composite leaf spring, which reduced the weight by 69.4% compared with the leaf spring. Through studying the mechanical properties of different composite materials, the most suitable material for preparing single-leaf spring was obtained. Al-Obaidi et al.^9^ studied the mechanical properties of composite materials for making leaf springs. The results show that the load-bearing capacity of composite leaf springs is related to the laying angle and volume content of the fiber, and the type of matrix has a significant effect on the stiffness. Nishant Varma et al.^10^ showed that the natural frequency of composite leaf spring is 93% higher than that of leaf spring. Chavhan et al.^11^ prepared E-glass fiber epoxy resin composite leaf spring, and studied its mechanical properties. The results showed that the strength of the composite leaf spring was close to the steel leaf spring, but the weight of the composite leaf spring was reduced by 79.13%. The authors introduce that composite leaf spring is simple to manufacture in the paper, but I think this conclusion is not rigorous. The manufacture of composite leaf spring should consider various factors such as lightweight, stiffness, strength, fatigue, deformation, etc. And the manufacturing process is also quite complicated, otherwise, the leaf spring sample can not be used in the actual vehicle. Umanath et al.^12^ introduced the manufacturing method of the leaf spring with carbon fiber and pineapple fiber as composite material. The strength and stiffness of the two composite leaf springs were compared in the paper. Before comparing the strength and hardness of different types of composite leaf springs, the fiber layup angle, volume content, and leaf spring stiffness should be controlled the same. At the same time, the fatigue performance is also an important performance of the leaf spring, and there is no comparative analysis in this paper. Rajendran et al.^13^ introduced that under the same design parameters and optimization conditions, 75.6% weight can be reduced by using single leaf spring instead of seven leaf spring. The deformation of the leaf spring has a great influence on the ride comfort and handling performance of the vehicle. When the authors optimized the composite leaf spring, the selected optimization targets were the weight, stiffness and strength of the leaf spring, and the deformation factor of the leaf spring should be considered at the same time. Hajime Kishi et al.^14^ introduced the preparation of composite laminates by vacuum pouring molding process, and compared the mechanical properties of glass fiber laminates with laying angles of ± 60° and ± 45°. Composite laminates are lightweight and thin-walled structures, and their damping characteristics are easily affected by the mass of the sensor and air damping. The authors should consider the above factors in the research process. The effects of different layering methods and chemical treatments on mechanical properties and free vibration properties of composites were discussed in literature^15^. Margherita Basso et al.^16^ described the nonlinear behavior of short glass fiber reinforced polymer composites through tensile creep test and stiffness degradation test.

Fiber reinforced composite leaf spring is composed of more than two kinds of polymer materials with different modulus and strength. By changing the content, type, direction and order of each component material, the composite spring with different mechanical properties can be obtained. However, due to the anisotropy and nonlinear characteristics of glass fiber reinforced composites^17^, it becomes difficult to study the dynamic characteristics of composite leaf springs theoretically.

Although there are many related studies, most of them focus on the theoretical analysis and finite element simulation analysis of the mechanical properties of composite leaf springs. There are few literatures on the experimental research on the mechanical properties of composite leaf springs. In this paper, the stiffness and damping properties of glass fiber resin matrix composite leaf spring were studied, and the effects of the fiber volume content and the laying angle on the stiffness and damping were studied in detail. The development of fiber reinforced composites in the field of automotive lightweight and suspension vibration reduction is promoted in this work.

## Materials and methods

### Materials and preparation

In this paper, PPG2026 glass fiber is selected as the reinforced material, and MAX2 polyurethane is selected as the matrix material. PPG2026 glass fiber belongs to E-glass fiber, produced by PPG Industries, USA. It has excellent mechanical properties and can form an excellent interface with the resin matrix to improve the fatigue performance of the composite leaf spring. MAX2 polyurethane has the characteristics of high strength, tear resistance, abrasion resistance, etc., and can form excellent interface bonding with glass fiber. The property parameters of materials are shown in Table 1.

**Table 1:** The property parameters of materials.

Parameter	Tensile strength (MPa)	Tensile modulus (MPa)	Density (g/cm^3^)	Elongation rate (%)
PPG2026	2650	82,700	2.58	3.2
MAX2	80	2800	1.12	7.5

**Figure 1:**
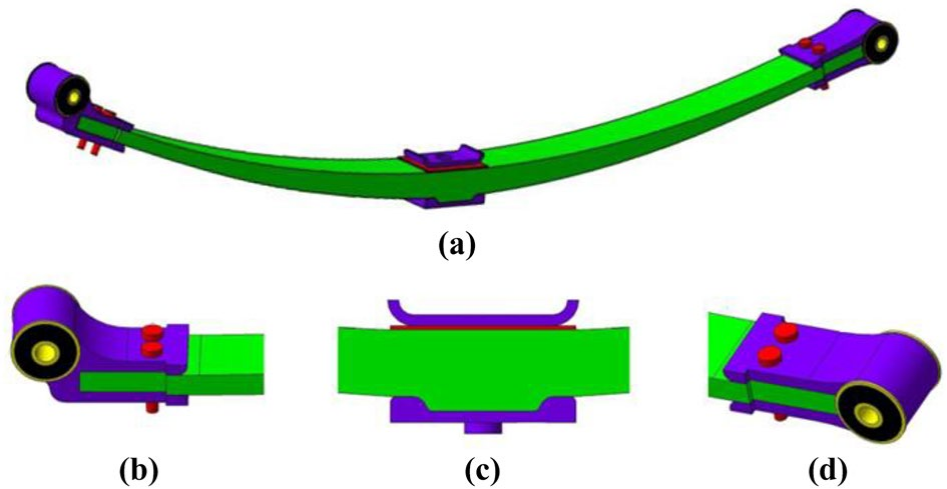
The composite leaf spring structure; (**a**) the 3D model of leaf spring; (**b**) the front connection structure; (**c**) the middle connection structure; (**d**) the rear connection structure.

The composite leaf spring is a multi-layer board structure bonded by multiple single-layer boards according to a specific laying angle and laying order. Its mechanical properties depend on the performance, content and laying angle of the glass fiber^18^. The volume content of glass fiber is an important performance parameter of E-glass fiber/polyurethane laminate. Too small fiber volume content cannot increase the effect. When the matrix strain is larger, the fiber will break. If the fiber volume content is too high, the strength of the composite material will be higher, but the fluidity of the resin will deteriorate, and the damping performance of the composite leaf spring will also decrease^19^. Comprehensively considering the mechanical properties of composite materials, the composite leaf springs with a laying angle of 0° were prepared, and the volume content of e-glass fiber was 40%, 60% and 80% respectively. According to the distribution of glass fibers in the matrix, composite laminates can be divided into unidirectional laminates and multidirectional laminates, and unidirectional laminates are fibers that are laid out in the same direction from multiple unidirectional plies. Multi-directional laminates are fibers made of multiple unidirectional plies laid in different directions^20^. The composite leaf springs with the fiber volume content of 60% were prepared, and the laying angle was 0°, 45° and 90°, respectively. The composite leaf spring structure is shown in Fig. 1a.

The composite leaf spring is composed of a leaf spring body, a front connection structure, a rear connection structure and a middle connection structure. The body of the leaf spring is a parabolic shape, and a boss structure is designed in the middle of the leaf spring body, and the boss structure is connected with the middle connecting structure to transmit longitudinal loads and lateral loads. Figure 1b is a front connection structure consisting of a front metal joint and a rubber bushing, which is connected to the front end of the leaf spring body through bolts. Figure 1c shows the middle connection structure, which consists of a lower metal plate, an upper metal plate and a U-bolt metal plate. Figure 1d is a rear connection structure consisting of a front metal joint and a rubber bushing, which is connected to the rear end of the leaf spring body through bolts.

In this paper, a high-pressure resin transfer molding process (HP-RTM process for short) is used to prepare E-glass fiber polyurethane composite leaf springs. The HP-RTM process uses high pressure to mix the resin and inject it into a vacuum sealed mold with fiber reinforced materials and preset inserts in advance. After the resin flow filling, impregnation, curing and demolding, the molding process of composite products is obtained^21,22^. In order to optimize the mechanical properties of the polyurethane resin, it is also necessary to use an oven to post-curing the leaf spring body. The final test specimens of composite leaf springs are shown in Table 2.

**Table 2:** Composite material leaf spring test specimens.

Specimen	Volume content/%	Laying angle/°	Length of leaf spring/mm	Width of leaf spring/mm	Number of layers
1#	60	0	1410	70	58
2#	60	45	1410	70	58
3#	60	90	1410	70	58
4#	40	0	1410	70	58
5#	80	0	1410	70	58

Due to the long working cycle and high cost of making composite leaf springs, it is difficult to make more samples with different laying angles and different volume contents. In order to obtain more data, the finite element model of composite leaf spring was established in this paper.

**Figure 2:**
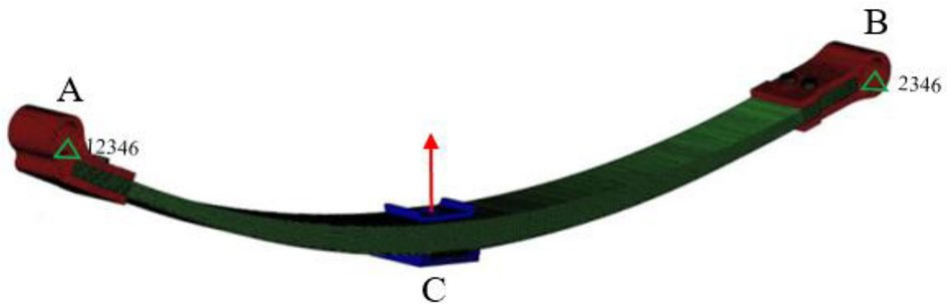
The finite element model of the composite leaf spring.

The 3D model of the composite leaf spring was imported into the HYPERMESH software, the model was divided into solid meshes, and the element type was C3D8I element. Figure 2 is a leaf spring finite element model with 457,482 elements and 518,750 nodes. The finite element model was imported into the ABAQUS software in the form of an INP file, and the material properties of the model were set according to the data in Table 1. The degree of freedom in the Ry direction of the rubber bushing A in the front connection structure is released, the degrees of freedom in the X and Ry directions of the rubber bushing B in the rear connection structure are released.

When simulating the static stiffness of the leaf spring, the z-direction displacement excitation is slowly applied to the middle connected structure C, and the static stiffness is calculated according to the force–displacement curve of the middle connected structure C. When simulating the dynamic stiffness of the leaf spring, a preload is applied to the leaf spring, and the Z-direction displacement amplitude is ± 10 mm, and the loading frequency is 4 Hz. When simulating the damping coefficient of the leaf spring, the transient Z-direction pulse displacement excitation is applied to the middle connected structure C, and the damping coefficient is calculated according to the acceleration time-domain curve of the middle connected structure C.

### Experimental method

**Figure 3:**
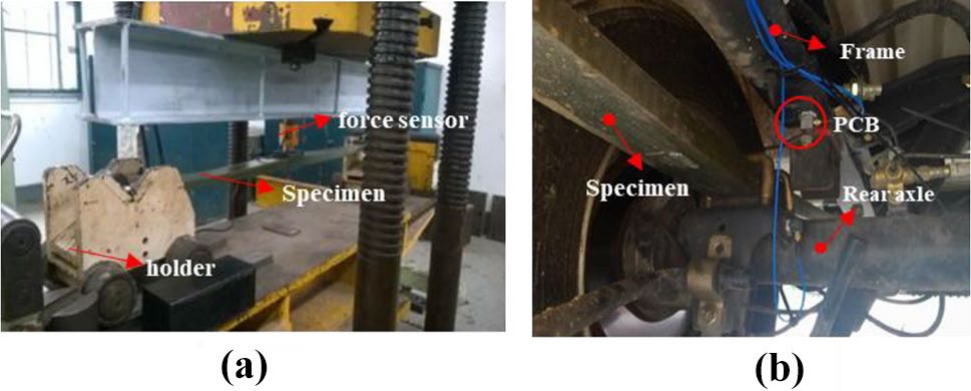
Mechanical property test of composite leaf spring; (**a**) the stiffness test; (**b**) the damping test.

The stiffness bench test of composite leaf spring is shown in Fig. 3a. The lugs at both ends of the composite leaf spring are respectively installed on the clamp respectively. When the leaf spring deforms, the clamp can be rolled along the extension direction of the leaf spring on the test-bed to simulate the actual working state of the compound leaf spring. The actuator of the bench is controlled by a hydraulic servo system. The load of the actuator during the test is collected by a force sensor in the middle of the leaf spring specimen, and the vertical displacement of the actuator during the test is measured by a vernier caliper. The slope of the load–displacement curve of the actuator is the stiffness of the composite leaf spring. The maximum dynamic deflection of the composite leaf spring designed in this paper is 140 mm. First, a vertical displacement is applied to the specimen through the actuator, the vertical displacement is gradually increased from 0 to 140 mm, and the vertical load of the actuator is recorded every 5 mm. Second, the vertical displacement imposed by the actuator on the specimen was gradually reduced from 140 to 0 mm, and the vertical load of the actuator was recorded every 5 mm.

Because the relative slip between glass fiber and polyurethane matrix consumes a lot of energy and the inherent viscoelasticity of the Matrix, the glass fiber polyurethane composite has good damping properties. The damping of the composite leaf spring mainly comes from the viscoelasticity of the composite material itself and the internal friction of the leaf spring. The damping is usually expressed by the damping coefficient. Due to the large stiffness of the composite leaf spring, its damping coefficient is difficult to be measured by the bench test method. In this paper, the leaf spring specimen is installed on the rear axle of the vehicle, and the damping characteristics of the specimen are tested by rolling down method. In order to avoid the impact of the shock absorber on the test results, the shock absorber in the rear suspension of the vehicle was removed before the damping test. The damping test uses the LMS TEST.LAB test system and the PCB three-way vibration acceleration sensor. The sensor is arranged on the frame above the rear axle of the vehicle, as shown in Fig. 3b. The acceleration signal of the vibration sensor on the frame is collected, and the damping coefficient of the compound leaf spring is calculated according to the amplitude attenuation ratio of the free vibration attenuation curve.

## Results and discussion

### Analysis of the influence of laying angle on stiffness

**Figure 4:**
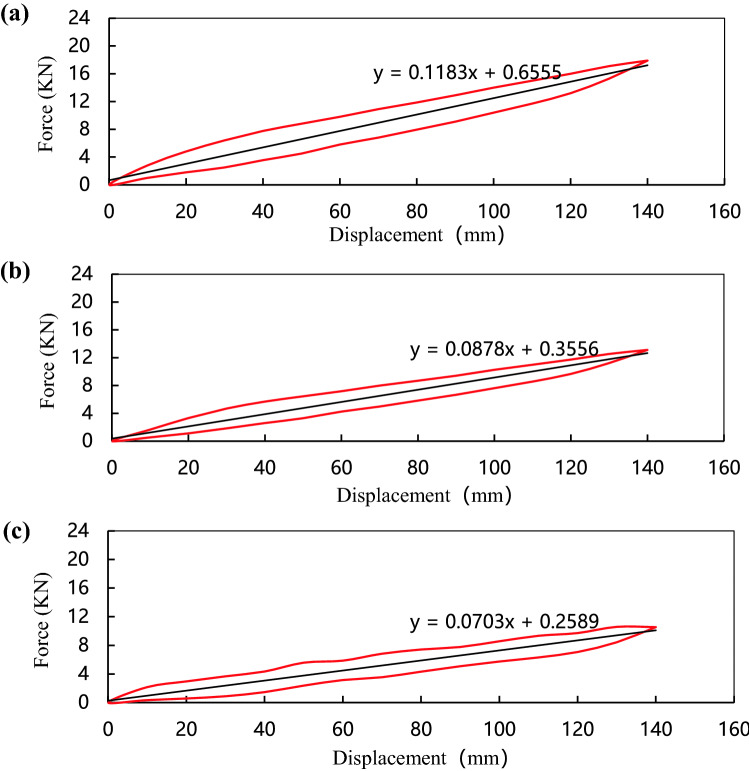
Stiffness curves of leaf springs with different laying angles; (**a**) Specimens 1 test data; (**b**) Specimen 2 test data; (**c**) Specimen 3 test data.

The stiffness of the composite leaf spring is determined by the mechanical properties of each single-layer plate, and affected by factors such as the volume content of the glass fiber, the number of layers, the laying angle, and the working temperature. In order to analyze the influence of the laying angle on the stiffness of the composite leaf spring, the specimens 1, 2 and 3 were selected for stiffness comparison tests. The stiffness test results are shown in Fig. 4.

Figure 4a–c show the stiffness curves of composite leaf springs with different laying angles when the glass fiber volume content is 60%. The curves show that the fiber laying angle has a significant effect on the stiffness of the composite leaf spring. The area enclosed by the hysteresis loop is the work done by the applied load, which decreases with the increase of the laying angle. Under the same displacement conditions, the larger the area, the greater the stiffness of the composite leaf spring. The stiffness of specimens 1, 2 and 3 are 118.3 N/mm, 87.8 N/mm and 70.3 N/mm, respectively. When the laying angle is 0°, the stiffness is maximum, when the laying angle is 90°, the stiffness is minimal.

**Figure 5:**
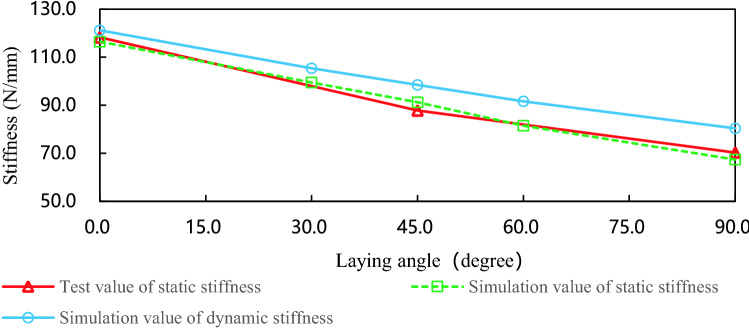
The simulation and experimental curves of leaf spring with different laying angles.

Figure 5 is the simulation and experimental curves of leaf spring with different laying angle. When the laying angle is 0°, the agreement between the test result of static stiffness and the simulation result of static stiffness is 98.5%, when the laying angle is 45°, the agreement is 96.3%, and when the laying angle is 90°, the agreement is 95.8%. The comparison results show that the finite element model of the composite leaf spring is correct. The dynamic stiffness of the leaf spring is greater than the static stiffness, and the difference between the two data increases with the increase of plying angle. Experimental data and simulation data show that when the volume content and the number of layers are constant, the greater the laying angle, the lower the stiffness, and it has a non-linear decreasing relationship with the laying angle.

### Analysis of the influence of glass fiber volume content on stiffness

**Figure 6:**
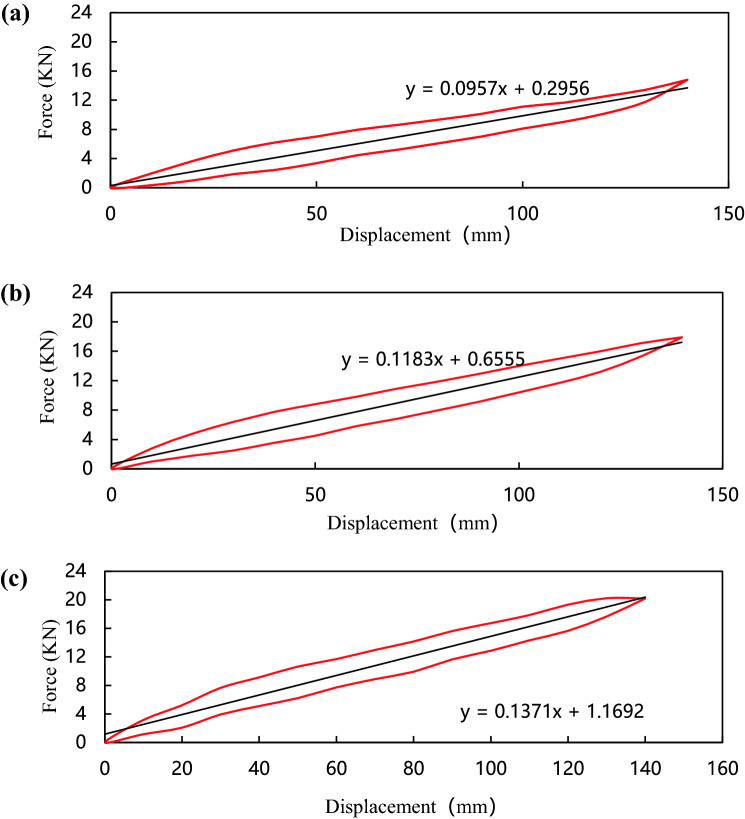
Stiffness curve of composite leaf spring with different fiber volume content; (**a**) specimen 4 test data; (**b**) specimen 1 test data; (**c**) specimen 5 test data.

For a composite leaf spring with a plying angle of 0°, the glass fiber volume content mainly affects the longitudinal tensile modulus. The higher the fiber volume fraction is, the higher the longitudinal tensile modulus is, and the greater the leaf spring stiffness is. In order to quantitatively analyze the relationship between the stiffness of the composite leaf spring and the volume content of glass fiber, specimens 1, 4 and 5 were selected for stiffness comparison tests. Figure 6a–c show the stiffness curves of composite leaf springs with different fiber volume content when the laying angle is 0°. The stiffness of specimens 4, 1 and 5 are 95.7 N/mm, 118.3 N/mm and 137.1 N/mm respectively. When the fiber volume content is 40%, the stiffness is minimal, when the fiber volume content is 80%, the stiffness is maximum.

**Figure 7:**
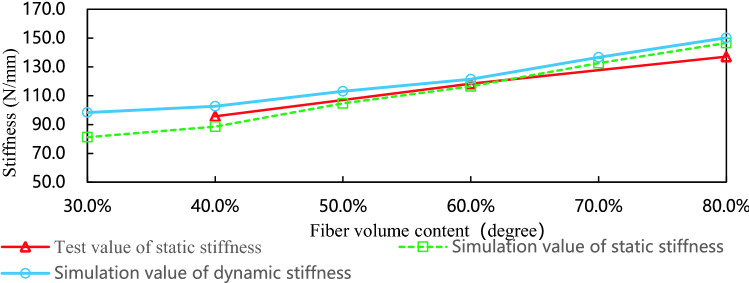
The simulation and experimental curves of leaf spring with different volume contents.

Figure 7 is the simulation and experimental curves of leaf spring with different volume content. When the volume content is 40%, the agreement between the test result of static stiffness and the simulation result of static stiffness is 92.5%, when the volume content is 60%, the agreement is 98.5%, and when the volume content is 80%, the agreement is 93.5%. The dynamic stiffness of the leaf spring is greater than the static stiffness, and the difference between the two data decreases with the increase of volume content. Experimental data and simulation data show that when the laying angle and the number of layers are constant, the larger the volume content, the higher the stiffness, and there is a linear increasing relationship with the fiber volume content.

### Analysis of the impact of laying angle on damping

**Figure 8:**
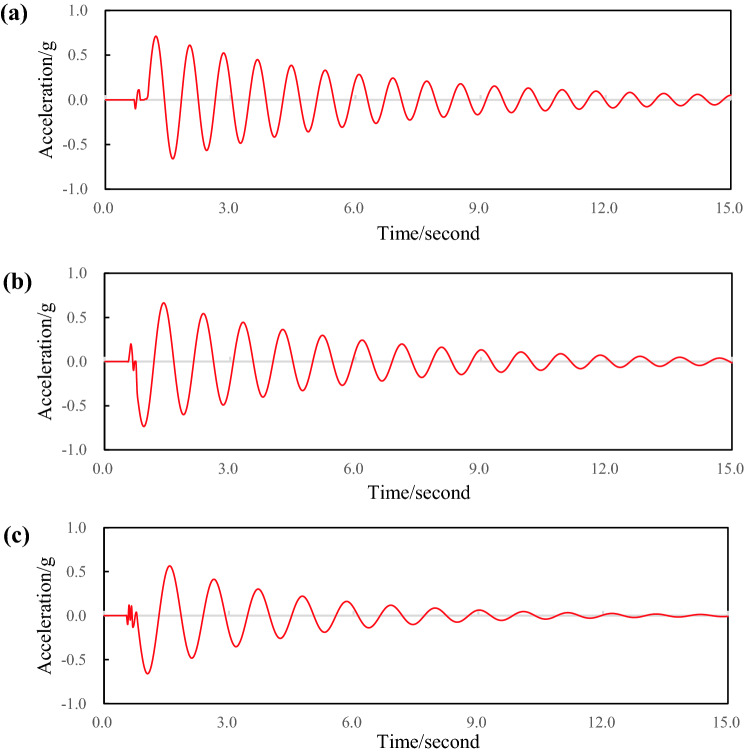
Variation curve of vibration attenuation of composite leaf springs with different laying angles; (**a**) specimen 1 test data; (**b**) specimen 2 test data; (**c**) specimen 3 test data.

The specimens 1, 2 and 3 were installed between the rear axle and the frame, and the shock absorbers of the rear suspension were removed, and the leaf spring damping test was carried out. Figure 8a–c show the free vibration attenuation curves of the composite leaf spring. The amplitude of adjacent cycles does not change much, which indicates that the damping of the composite leaf spring is small. The damping coefficients of specimens 1, 2 and 3 are 0.024, 0.031 and 0.044 respectively. The damping coefficient of the leaf spring with 0° layer is the smallest, and the damping coefficient of the leaf spring with 90° layer is the largest.

The fiber laying angles affect the overall stiffness, inter-laminar friction and shear properties of laminates. When the stiffness of composite leaf spring is too large, the deformation ability of polyurethane will be affected, and the relative sliding between the reinforcement and the matrix will be impeded, thus reducing the damping characteristics of composite leaf spring^23,24^. Specimen 1 is laid at 0°, the fiber layer plays the main bearing role, and the stiffness of the leaf spring is the largest. Under the same external load, the composite leaf spring consumes less vibration energy and has the smallest damping coefficient. Specimen 5 is laid at 90°, and the polyurethane layer plays the main bearing role, resulting in the lowest stiffness of the leaf spring. Under the same external load, the composite leaf spring consumes more vibration energy and has the largest damping coefficient.

**Figure 9:**
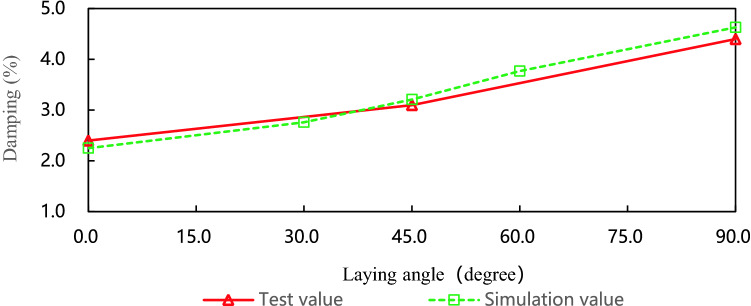
The damping contrast curves of leaf springs with different laying angles.

Figure 9 is the damping contrast curves of leaf spring with different laying angles. When the laying angle is 0°, the agreement between the test result and the simulation result is 93.8%, when the laying angle is 45°, the agreement is 96.6%, and when the laying angle is 90°, the agreement is 95.0%. Experimental data and simulation data show that when the fiber volume content and the number of layers are constant, the larger the laying angle, the greater the damping coefficient of the composite leaf spring, and it has an approximate linear growth relationship with the laying angles.

### Analysis of the influence of glass fiber volume content on damping

**Figure 10:**
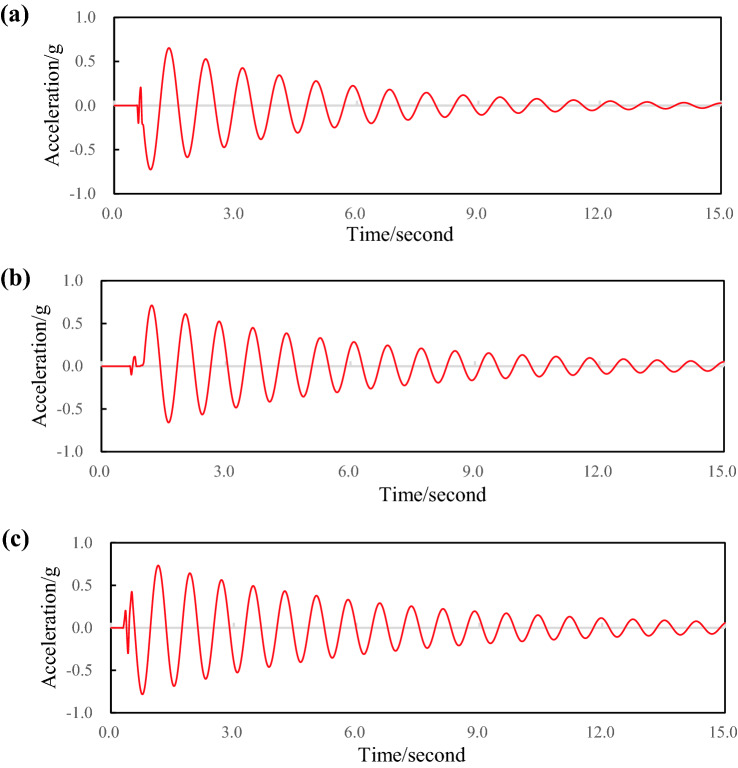
Vibration attenuation curve of composite leaf spring with different volume contents; (**a**) specimen 4 test data; (**b**) specimen 1 test data; (**c**) specimen 5 test data.

The specimens 1, 4, 5 were selected for damping tests. Figure 10a–c show the free vibration attenuation curves of the composite leaf spring specimens. The damping coefficients of specimens 4, 1 and 5 are 0.032, 0.024 and 0.0197, respectively. When the fiber volume content is 40%. The damping coefficient of the leaf spring is the largest. When the fiber volume content is 80%, the damping coefficient of the leaf spring is the smallest.

The main contribution of the composite leaf spring damping comes from the polyurethane matrix. Polyurethane is viscoelastic. When a force is applied to the leaf spring, the polyurethane matrix will undergo tensile deformation, bending deformation and shear deformation, which consumes vibration energy to achieve the vibration reduction effect. The higher the glass fiber volume content, the lower the polyurethane content, the worse the viscoelasticity of the composite leaf spring and the lower the damping coefficient.

**Figure 11:**
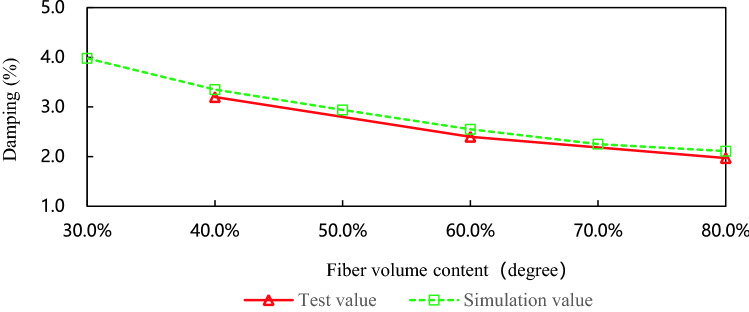
The damping contrast curves of leaf springs with different volume contents.

Figure 11 is the damping contrast curves of leaf springs with different volume contents. When the volume content is 40%, the agreement between the test result and the simulation result is 95.5%, when the volume content is 60%, the agreement is 94.1%, and when the volume content is 80%, the agreement is 93.4%. Experimental data and simulation data show that when the laying angle and the number of layers are constant, the higher the fiber volume content, the smaller the damping coefficient. However, when the fiber volume content increases to a certain extent, the influence of the fiber volume content on the damping coefficient of the composite leaf spring will become insignificant.

## Conclusion

When the fiber volume content and number of layers of the composite leaf spring remain unchanged, the larger the laying angle, the smaller the stiffness and the larger the damping coefficient.

When the fiber laying angle and number of layers of the composite leaf spring remain unchanged, the larger the fiber volume content, the larger the stiffness and the smaller the damping coefficient.

The finite element model of the composite leaf spring is established in the paper, and the stiffness and damping are simulated and analyzed. The simulation data is in good agreement with the experimental data. The finite element simulation method is helpful to study the mechanical properties of the leaf spring.

The research results of the stiffness and damping of composite leaf springs provide a theoretical basis and reference for the performance design of leaf springs.

## Data Availability

All data generated or analysed during this study are included in this published article.
